# Multimodal Prediction of Alzheimer's Disease Severity Level Based on Resting-State EEG and Structural MRI

**DOI:** 10.3389/fnhum.2021.700627

**Published:** 2021-09-09

**Authors:** Belmir Jesus, Raymundo Cassani, William J. McGeown, Marco Cecchi, K. C. Fadem, Tiago H. Falk

**Affiliations:** ^1^Institut National de la Recherche Scientifique, University of Quebec, Montreal, QC, Canada; ^2^School of Psychological Sciences and Health, University of Strathclyde, Glasgow, United Kingdom; ^3^COGNISION, Louisville, KY, United States

**Keywords:** Alzheimer's disease, electroencephalography, magnetic resonance imaging, AD severity, multimodal prediction

## Abstract

While several biomarkers have been developed for the detection of Alzheimer's disease (AD), not many are available for the prediction of disease severity, particularly for patients in the mild stages of AD. In this paper, we explore the multimodal prediction of Mini-Mental State Examination (MMSE) scores using resting-state electroencephalography (EEG) and structural magnetic resonance imaging (MRI) scans. Analyses were carried out on a dataset comprised of EEG and MRI data collected from 89 patients diagnosed with minimal-mild AD. Three feature selection algorithms were assessed alongside four machine learning algorithms. Results showed that while MRI features alone outperformed EEG features, when both modalities were combined, improved results were achieved. The top-selected EEG features conveyed information about amplitude modulation rate-of-change, whereas top-MRI features comprised information about cortical area and white matter volume. Overall, a root mean square error between predicted MMSE values and true MMSE scores of 1.682 was achieved with a multimodal system and a random forest regression model.

## 1. Introduction

Alzheimer's disease (AD) is a neurodegenerative condition that progressively affects cognitive functioning by impairing nerve cell function in the brain (Gross et al., 2016). This process may start 20 years or more before symptoms become evident (Alzheimer's Association, 2019). The progression of AD negatively influences the lives of people living with the disease, their families, and their caregivers, since the pathology is linked to decline or loss of mental abilities, which are essential for many daily activities (Qiu et al., 2017).

One topic of intense research focus has been on improving early diagnosis of Alzheimer's disease. Whilst definitive diagnosis of AD is currently only achievable with postmortem neuropathological examination, *in vivo* diagnosis is often based on clinical criteria, which rely on clinical interview with support from cognitive assessments. Global cognitive assessment is often accomplished using paper and pencil tests such as the Montreal Cognitive Assessment (MoCA) (Nasreddine et al., 2005) or the Mini-Mental State Examination (MMSE) (Folstein et al., 1975). Given the co-occurrence of neuropathological and structural brain changes that occur alongside cognitive impairment, research has also focused on developing biomarkers of the disease that may help to increase confidence in the diagnosis. Over the last two decades, a number of biomarkers have been proposed, including those based on structural neuroimaging (e.g., hippocampal volume from magnetic resonance imaging) (Chetelat and Baron, 2003; Frisoni et al., 2010), cerebral spinal fluid (e.g., tau and beta-amyloid levels) (Caroli et al., 2010), blood and urine samples (Mayeux and Schupf, 2011), electroencephalography (Cassani et al., 2018), and more recently, genetic risk profiling (Van Cauwenberghe et al., 2016). Given that changes in biomarkers can also precede clinical symptoms and overt cognitive impairment, attempts have been made to use certain biomarker measures (e.g., amyloid deposition and hippocampal atrophy) to identify people who are at risk of developing dementia in the future and for whom disease modifying therapies may be suitable. It is thought that disease-modifying therapeutic interventions should be most effective when administered in the early stages of the disease, before neuronal loss occurs (Jedynak et al., 2012), thus early detection is crucial.

A research topic that has received relatively less attention is that which aims to determine biomarker features that are associated with increasing disease severity. With the availability of such biomarker features, healthcare professionals and clinical trial teams would have additional tools to monitor the disease. Relying upon cognitive assessment only may be problematic, as performance on tasks such as the MMSE may be subject to confounding variables, such as stress levels (e.g., Freidl et al., 1996) or sleep duration (e.g., Ramos et al., 2013). Importantly, when measured across multiple time-points, cognitive tests such as the MMSE are also prone to practice effects (e.g., Galasko et al., 1993) that may mask any decline, whereas biomarkers avoid this issue. Biomarkers may offer measurement along multiple dimensions, and some biomarkers, for example, those that utilize electroencephalography (EEG), might also have analogs that can be adopted in drug trials using animal models, where assessment of some cognitive functions (e.g., language, visuoconstruction) would not be possible. As more therapies, drugs, and interventions appear, monitoring the progression and slowing of the disease will become crucial to gauge the benefits of the different interventions (Wild et al., 2008). Monitoring change may be particularly challenging when confined to the early stages of disease, as symptoms may not be as pronounced, and knowledge of subtle changes to biomarkers will therefore be relevant.

In relation to monitoring disease progression, in Jedynak et al. (2012), for example, several biomarkers from the Alzheimer's Disease Neuroimaging Initiative (ADNI) database have been suggested, such as hippocampal volume, tau and beta-amyloid levels. In Eskildsen et al. (2015), in turn, hippocampal atrophy and cortical thickness of a selection of temporo-parietal regions were shown to be useful predictors of mild cognitive impairment progressing to AD. More recently, in Cassani et al. (2018), it was shown that only a few studies have explored the effects of disease progression on EEG. In particular, earlier work showed that changes in specific frequency subbands could be seen with disease progression (e.g., Kuskowski et al., 1993; Kowalski et al., 2001). More recently, spectrotemporal energy patches were shown to provide improved accuracy (relative to conventional subband powers) when predicting disease severity levels (Cassani and Falk, 2020).

As AD manifests itself across different facets, it is expected that a single biomarker may not provide sufficient information to determine disease severity accurately (Gross et al., 2016) and that instead, a multimodal biomarker system would be needed (Hampel et al., 2010). Despite multimodal biomarkers being explored for the detection of AD and mild cognitive impairment (MCI) (Zhang et al., 2011), research is again limited on multimodal systems for AD severity detection (Martínez-Torteya et al., 2015). Moreover, of those studies that have investigated multimodal biomarkers for early detection, most have relied on structural neuroimaging tools (Falahati et al., 2014) and only a handful have explored the fusion of structural neuroimaging with electro-neurophysiology (Cassani et al., 2018). This paper aims to fill these gaps, by investigating multimodal biomarker features that are associated with increasing disease severity, and demonstrate that EEG tests, with minimal interventions from the participants (i.e., resting state 3-min recordings) and a semi-automated processing pipeline, could further improve the accuracy of a disease severity monitoring system relying on neuroimaging information.

Focusing on the fusion of electrophysiological and structural neuroimaging features should have utility in predicting the severity of the disease, and in identifying useful markers for monitoring disease progression, as together they contribute information on neuronal injury, atrophy and synaptic integrity. Considering the amyloid-tau-neurodegeneration (A/T/N) biomarker framework proposed in Jack et al. (2016), electrophysiological and structural magnetic resonance imaging (MRI) measures can provide information on the neurodegeneration/neuronal injury (N) aspect of disease progression (with structural MRI mentioned explicitly as a recommended technique in the framework). To measure disease severity, we chose to use the MMSE due to its widespread use in clinical practice. In this test, a clinician asks the patient several questions about daily mental competencies to evaluate their cognitive state. The participant is then given a score based on their answers, with a maximum of 30 points. Often scores of 20–24 are taken to be indicative of mild dementia, scores of 13–20 suggest moderate dementia, and those less than 12 are indicative of severe dementia. The MMSE scores of the patients in our sample were subsequently used to derive the multimodal features (MRI and EEG) that could also be used to track disease severity.

In preview, this paper reports the development of multimodal markers of AD severity for patients in the early stages of the disease (minimal to mild dementia; MMSE scores between 21 and 26). We explore the fusion of cortical, subcortical and white matter features extracted from MRI, as well as conventional EEG features alongside ones recently developed, such as the spectrotemporal energy patch features (Cassani and Falk, 2020). Analyses on data from 89 patients show the usefulness of multimodal AD severity level models for patients at these stages of AD, and access to an automated tool could be useful for clinicians to help with better diagnostics and monitoring.

## 2. Materials and Methods

### 2.1. Participants

The data used herein was collected from a multi-site clinical trial exploring the use of electrophysiological markers to study Alzheimer's disease (Cecchi et al., 2015). Initial recruitment included 103 subjects with probable AD and 101 healthy controls. The study was approved by the institutional review boards for each trial site and consent was obtained from each participant. The interested reader is referred to Cecchi et al. (2015) for more details about inclusion and exclusion criteria, as well as all the tests and criteria performed for diagnostics.

Here, we were particularly interested in the target population from whom cognitive data, resting-state EEG and structural MRI scans were all available. As MRI scans were only available for the patients with AD, the healthy controls were excluded from our analyses. EEG data was available for 99 of the AD patients, and of these, 5 did not have MRI scans. After further careful screening for sufficient scan quality to enable accurate MRI segmentation/parcellation (e.g., removing scans with motion/ringing artifacts), five more participants were excluded. This left data from a total of 89 (46 females) participants for the analyses. The participants had an average age of 75.8 ± 7.3 years and average of 14.5 ± 3.3 years of education. All patients had MMSE scores between 21 and 26, with an average of 23.3 ± 1.8.

### 2.2. Semi-automated Analysis Pipeline

**Figure 1:**
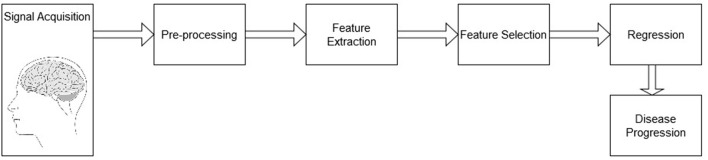
Block diagram of the processing pipeline for AD severity level prediction.

The system analysis pipeline follows the diagram depicted by Figure 1. The pipeline is comprised of a signal acquisition step, followed by pre-processing, feature extraction, feature selection, and finally a regression mapping to predict disease severity level. Here, the main goal is to predict the patient's MMSE score based on EEG features alone, MRI features alone, as well as with a combined multimodal feature set. The next sections details these steps.

### 2.3. Signal Acquisition

**Figure 2:**
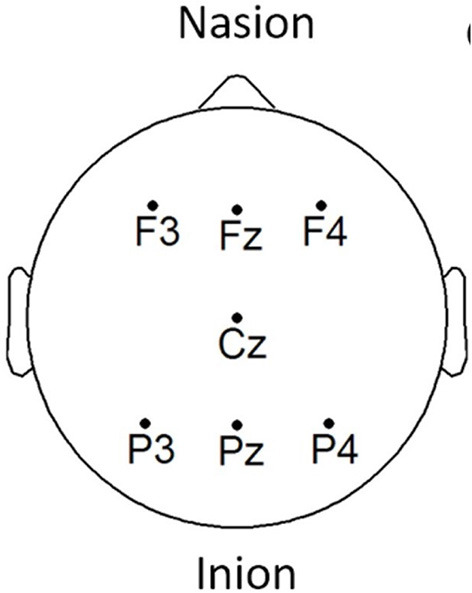
EEG electrode placement, adapted from Cassani et al. (2017).

EEG signals were acquired using the COGNISION^®^ device (Casey, 2010), a seven-channel device operating at a sampling frequency of 125 Hz. Bi-auricular referential electrodes were also attached during the collection procedure. For each of the participants, we are interested in the EEG collected during a 3-min resting awake eyes-open period. Figure 2 displays the placement of the EEG electrodes located at the F3, Fz, F4, Cz, P3, Pz, and P4 locations. Motivated from Falk et al. (2012), two virtual inter-hemispheric bipolar signals are also used, namely: F3-F4 and P3-P4.

Depending on the scanning site (*n* = 6), 1.5 Tesla or 3 Tesla scanners were used. T1-weighted MRI scans were acquired for the structural analyses. After the exclusions due to artifacts, 24 scans remained for the analysis at 1.5 Tesla and 65 at 3 Tesla. Varying across scanning site, slice thickness ranged from 1-1.2 mm, and dimensions in the axial plane from 0.5 to 1.25 mm^2^. In the set of scans used for the analyses no movement artifacts were visible, nor were any artifacts thought to affect segmentation/parcellation.

### 2.4. Pre-processing

EEG signals were first pre-processed through a zero-phase finite impulse response (FIR) band-pass filter with a bandwidth of 0.5–45 Hz to eliminate any power grid measurement interference. Moreover, motivated by the results presented in Cassani et al. (2014a), a wavelet-independent component analysis (wICA) was further applied to remove unwanted ocular and muscular artifacts. The interested reader is referred to Mammone et al. (2011) for more details about this EEG artifact removal algorithm.

The T1-weighted MRI scans, in turn, were processed using FreeSurfer^1^, a universally used open-access software package to process and analyze structural brain MRI scans that has been developed and validated by MRI researchers (Fischl, 2012). The images captured from the MRI scanner were pre-processed using the default command “recon-all” provided in the FreeSurfer (v6) pipeline. The “recon-all” procedure, when used with the “-all” flag instructs FreeSurfer to perform its full reconstruction pipeline, which provides subcortical segmentation and volume measurement, in addition to cortical reconstruction, and measurement of a set of regions of interest (thickness and area). For the pre-processing of the 3 Tesla scans, the additional “-3T” flag was applied, and the “-mprage” flag was applied where appropriate. Aiming for automaticity, reliability and reproducibility, the pipeline was applied without manual intervention. Although there may be some benefits to measurement accuracy through a semi-automated approach with manual edits, the fully automated pipeline has been demonstrated to be consistent with manual measurements made both in and *ex vivo* (e.g., Fischl et al., 2002; Cardinale et al., 2014). Briefly, the FreeSurfer pre-processing pipeline involves non-uniform intensity normalization, Talairach transform computation, intensity normalization, skull stripping, subcortical segmentation (including volumetric labeling and measurement), intensity normalization (this time with the brain volume as the input, in the absence of the skull), white matter segmentation, tessellation (of the gray and white matter boundary), automatic topology correction, creation of final surfaces (pial and white matter), and parcellation (creation and measurement). Key papers that cover the methodology underpinning the pre-processing pipeline can be found in Dale et al. (1999), Fischl et al. (1999), Fischl et al. (2002), and Fischl et al. (2004).

### 2.5. Feature Extraction

#### 2.5.1. EEG Features

In the context of Alzheimer's disease, four different features were computed from the acquired EEG signals based on insights from Cassani et al. (2017) and Cassani and Falk (2020). These included: spectral power, magnitude squared coherence, amplitude modulation rate-of-change, and modulation frequency “patches” features. Features were extracted over 8-s epochs with 1-s shifts between consecutive epochs.

##### 2.5.1.1. Spectral Power

Spectral power features are defined by the power measurements within each of the following EEG frequency subbands: delta (0.5–4 Hz), theta (4–8 Hz), alpha (8–12 Hz), low-alpha (8–10 Hz), high-alpha (10–12 Hz), beta (12–30 Hz), delta-to-beta (0.5–30 Hz), theta-to-beta (4–30 Hz), and low-gamma (30–45 Hz). To calculate the spectral power features, zero-phase FIR bandpass filters were used to decompose the EEG signal into different frequency bands of interest. Then, power is computed for each different subband time series. Next, normalization was performed by dividing the subband power by the full-band EEG power. A total of 81 spectral power features were computed, corresponding to the nine frequency bands, per seven electrode locations plus the two virtual inter-hemispheric channels.

##### 2.5.1.2. Coherence

Here, the magnitude squared coherence (MSC) feature is used to measure the co-variance between two power spectra. Coherence features were computed for delta, theta, alpha, beta, and gamma bands for five electrode connections, namely: Fz-Pz, F3-F4, P3-P4, F3-P3, and F4-P4. A total of 25 features were calculated for this type of feature (5 frequency bands × 5 pairs of electrodes).

##### 2.5.1.3. Amplitude Modulation Rate-of-Change

**Figure 3:**
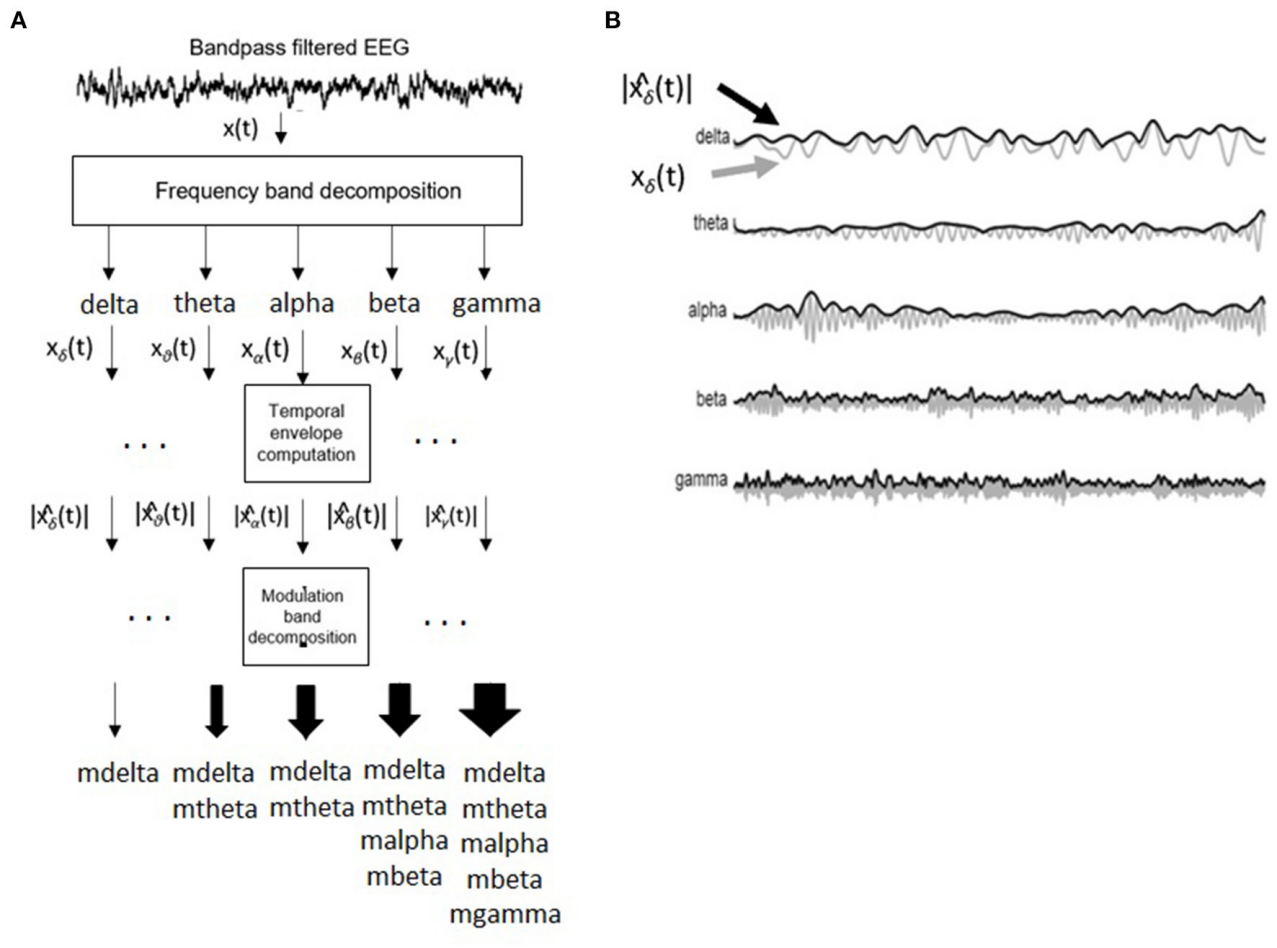
**(A)** Signal processing steps involved in computation of amplitude modulation rate-of-change features. **(B)** Bandpass filtered EEG signals and their time envelopes. Adapted from Falk et al. (2012).

Figure 3 depicts the signal processing steps involved in the computation of the amplitude modulation rate-of-change features proposed in Falk et al. (2012). To compute the amplitude modulation features, the EEG signal is first decomposed in the delta, theta, beta, and gamma bands. Next, the Hilbert transform is applied to compute each subband envelope. A second frequency decomposition is then applied to the band envelope signals to identify the changes in their amplitude modulation. Lastly, the average energy per frequency-modulation band is computed, normalized by the total signal energy, and used as a feature. Given properties of the Hilbert transform, not all frequency-modulation frequency combinations are possible, thus the following combinations were used: delta-mdelta, theta-mdelta, theta-mtheta, alpha-mdelta, alpha-mtheta, beta-mdelta, beta-mtheta, beta-malpha, beta-mbeta, gamma-mdelta, gamma-mtheta, gamma-malpha, gamma-mbeta, and gamma-mgamma. Hence, a total of 126 features were extracted (14 frequency-modulation bands × 9 electrodes) to quantify amplitude modulation spectra rate-of-change.

##### 2.5.1.4. Modulation Frequency “Patches”

**Figure 4:**
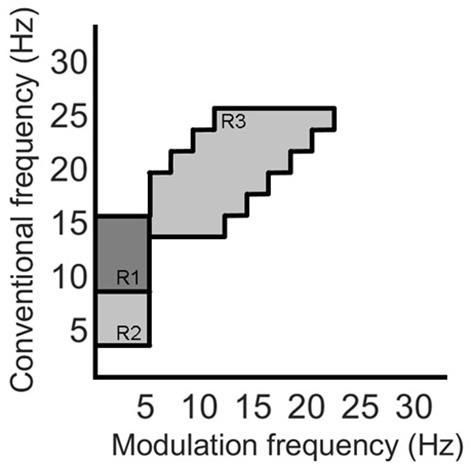
Three regions (patches) identified in the modulation spectral domain to provide best AD diagnostic accuracy, adapted from Cassani and Falk (2020).

Recently, the work by Cassani and Falk (2020) showed that improved AD diagnostics could be achieved if non-conventional bands were used in the calculation of the above-mentioned amplitude modulation rate-of-change features. These so-called “patches,” as seen in Figure 4, were shown to be important in discriminating mild cognitive impairment from AD, as well as moderate AD from severe AD. A total of three patches (termed R1-R3 in the figure) and their ratios provided the most discriminatory information. Here, a total of 54 features are extracted corresponding to the three patches and their ratio combinations for each of the seven electrodes and two virtual bipolar channels. Overall, a total of 286 features were extracted from each of the 8-s epochs. Table 1 summarizes the features calculated for each EEG epoch.

**Table 1:** Summary of the features computed from EEG signals.

**Features category**	**Features**
Spectral power	delta
	theta
	alpha
	low-alpha
	high-alpha
	beta
	delta-beta
	theta-beta
	gamma
Coherence	MSC: delta
	MSC: theta
	MSC: alpha
	MSC: beta
	MSC: gamma
Amplitude modulation rate-of-change	delta-mdelta
	theta-mdelta
	theta-mtheta
	alpha-mdelta
	alpha-mtheta
	beta-mdelta
	beta-mtheta
	beta-malpha
	beta-mbeta
	gamma-mdelta
	gamma-mtheta
	gamma-malpha
	gamma-mbeta
	gamma-mgamma
Modulation frequency-patches	R 1
	R 2
	R 3
	R 1 /R 3
	R 2 /R 1
	R 2 /R 3

#### 2.5.2. EEG Feature Functionals

As mentioned above, EEG features are computed per time epoch. MRI features, in turn, are available per subject. As such, in order to enable feature-level fusion experiments, we must aggregate all EEG features into one final per-subject feature. Here, summary statistics are used for this purpose. More specifically, mean, standard deviation, coefficient of variation, median, skewness, and kurtosis were obtained for each of the four EEG feature types (spectral power, coherence, amplitude modulation, and modulation patches) (Devore, 2008). In the end, a total of 1716 EEG features exist corresponding to the 286 per-epoch features times their six summary statistics.

#### 2.5.3. MRI Feature Extraction

As described above, the MRI features were extracted using the FreeSurfer software package. Subcortical volumes, cortical thickness (CT), surface area, and white matter volume measures were extracted from different regions-of-interest (*ROIs*). The interested reader is referred to Fischl et al. (2002) and Fischl et al. (2004) for more details on the Freesurfer methods for feature extraction.

##### 2.5.3.1. Subcortical Segmentation

Subcortical segmentation of the brain volumes were performed using the automatic subcortical segmentation provided in FreeSurfer. Segmentation based on the ASEG atlas was used, which locates and labels the structure using probabilistic means (Fischl et al., 2002, 2004). A detailed list of the volumes of anatomical structures that were included in the study is specified in the Supplementary Materials. A total of 62 features of subcortical segmentation measurements were available.

##### 2.5.3.2. Cortical Parcellation

Average thickness and surface area of the cortical parcellation were calculated. To this end, the MRI scans were parcellated into distinct anatomical regions, according to the boundaries and labeling of the Desikan-Killiany atlas (Desikan et al., 2006). Cortical thickness measures were taken as the distance between the boundary between the white matter/gray matter and the pial surface (in mm), and these were averaged for each ROI (Fischl and Dale, 2000). Surface area for each ROI was calculated (in mm^2^). Measurements of cortical structure were determined for the left and right hemisphere ROIs, and a list with the measurements considered for the study is added in the supplementary materials. A total of 148 features were computed for cortical parcellation measurements.

##### 2.5.3.3. White Matter Parcellation

White matter parcellation was performed based on the labeling provided by the Desikan-Killiany atlas. White matter volumes were calculated based on proximity to the cortical label, and a constraint in the form of an extension of 5 mm into the white matter (Salat et al., 2009). White matter volume measures were estimated for the left and right hemispheres, and the list of ROIs utilized is presented in the supplementary materials. There were a total of 75 features resulting from the white matter parcellation process.

Table 2 summarizes the 285 features calculated for each of the MRI scans. Subsequently, all the calculated features (both EEG and MRI based) were submitted to a normalization process. Normalization was utilized to scale all the features in a comparable range from the value of –1 to 1 and avoid future problems with regression models that could be dependent on distance calculations. Each individual feature were scaled by its maximum absolute value.

**Table 2:** Summary of the MRI computed features.

**Features category**	**Features**
Subcortical segmentation-ASEG	62 volume measurements of anatomical structures provided by the subcortical segmentation.
Cortical parcellation-APARC	148 thickness and area measurements of anatomical structures resulted from the cortical parcellation.
White matter parcellation-WM	75 volume measurements of anatomical structures resulted from the white matter parcellation.

### 2.6. Feature Selection

At the end of the feature extraction procedure, a total of 1,716 features were calculated from the EEG signals and a total of 285 features were calculated from MRI scans. While our main goal is to explore the benefits of a multimodal system, one exploratory aim we have is to also gauge the benefits of one single modality over the other. In the multimodal system, we use the feature fusion technique to combine modalities. In our experiments, feature fusion is achieved by concatenating the feature matrices into one final larger feature matrix. Given the large number of features and the limited size of the available dataset, feature selection is needed to reduce the number of features to avoid overfitting (Jović et al., 2015). Feature selection or ranking aims to rank available features based on their potential impact on the downstream classification task (Cai et al., 2018). This way, irrelevant and/or redundant features are removed.

Many feature selection techniques have been reported in the literature, and there is no consensus on what technique is ideal for a specific application, hence different methods are commonly explored. Here, different univariate filter techniques for feature selection are investigated. Filter techniques do not utilize any learning model during the feature selection process, thus, the ranking is based solely on data characteristics (Bagherzadeh-Khiabani et al., 2016). Combined with a univariate approach, the feature selection step evaluates each feature in the dataset one-by-one by determining their relationship with the dependent variable, or label to be predicted; in our case, MMSE scores. In the end, the features are ranked based on this relationship and the features with the highest scores are selected. In this study, three different techniques of filtering the features were tested: Pearson correlation, Spearman correlation, and Minimum Redundancy Maximum Relevance (MRMR).

#### 2.6.1. Correlation-Based Ranking

Correlation-based feature selection relies on correlation measures between the tested features and the outcome variables. Different types of correlations are available to measure different associations between the two variables. Here, two of the most popular indices of correlation are used, namely Pearson and Spearman correlations. Pearson correlation ranges from –1 to 1 and measures a linear relationship between the variables, with a correlation coefficient of zero indicating no linear relationship. Spearman correlation, in turn, measures the ranking relationship between variables, thus considers linear relationships and non-linear monotonic relationships between variables (Chok, 2010). As mentioned by Cassani et al. (2018), correlation-based methods are widely used in EEG-based AD diagnostic systems.

#### 2.6.2. MRMR

One main drawback of correlation-based methods is that while keeping relevant features, their redundancy is not accounted for, thus multiple features may be kept while providing limited additional information for the downstream classification task (Dormann et al., 2013). As such, it may be seen as suboptimal. To compensate for this limitation, the minimum redundancy maximum relevance (MRMR) feature selection algorithm has been proposed. In essence, the algorithm selects a subset of features having the most relation with a class (relevance) and the least relation between themselves (redundancy). Relevance can be calculated with an F-statistic metric or mutual information, whereas redundancy can be calculated by using Pearson correlation coefficient or mutual information (Peng et al., 2005; Li et al., 2017; Cai et al., 2018). For our usage, an open-source implementation of the MRMR was used (Li et al., 2017).

### 2.7. Top-Feature Grouping

In an attempt to match the dimensionality reported in previous EEG-based AD literature (e.g., Falk et al., 2012; Fraga et al., 2013; Cassani et al., 2014b), the top 24 features from each of the feature modalities was selected. Here, we call feature “Group 1” the top-24 features selected for the EEG modality. Group 2, in turn, corresponds to the top-24 MRI features. As we are particularly interested in multimodal systems, two other feature fusion strategies are explored. First, we apply feature selection to the fused EEG-MRI features sets and keep the top 24 features of this combined set. This is termed feature Group 3. Earlier work has suggested some correlation between EEG and MRI features (McGeown et al., 2019), thus performing feature selection of the fused set may help remove redundant features from varying modalities. Lastly, we combine the top-12 EEG and top-12 MRI features and call this feature Group 4. Table 3 summarizes the four top feature groups explored herein.

**Table 3:** Top-feature grouping classes.

**Groups**	**Description**
Group 1	Top 24 features from the ranking of features from EEG signals.
Group 2	Top 24 features from the ranking of features from MRI scans.
Group 3	Top 24 features from the ranking of the combination of features from EEG and MRI.
Group 4	Combination of Top 12 features from group 1 with the top 12 features from group 2.

### 2.8. Data Partitioning

**Figure 5:**
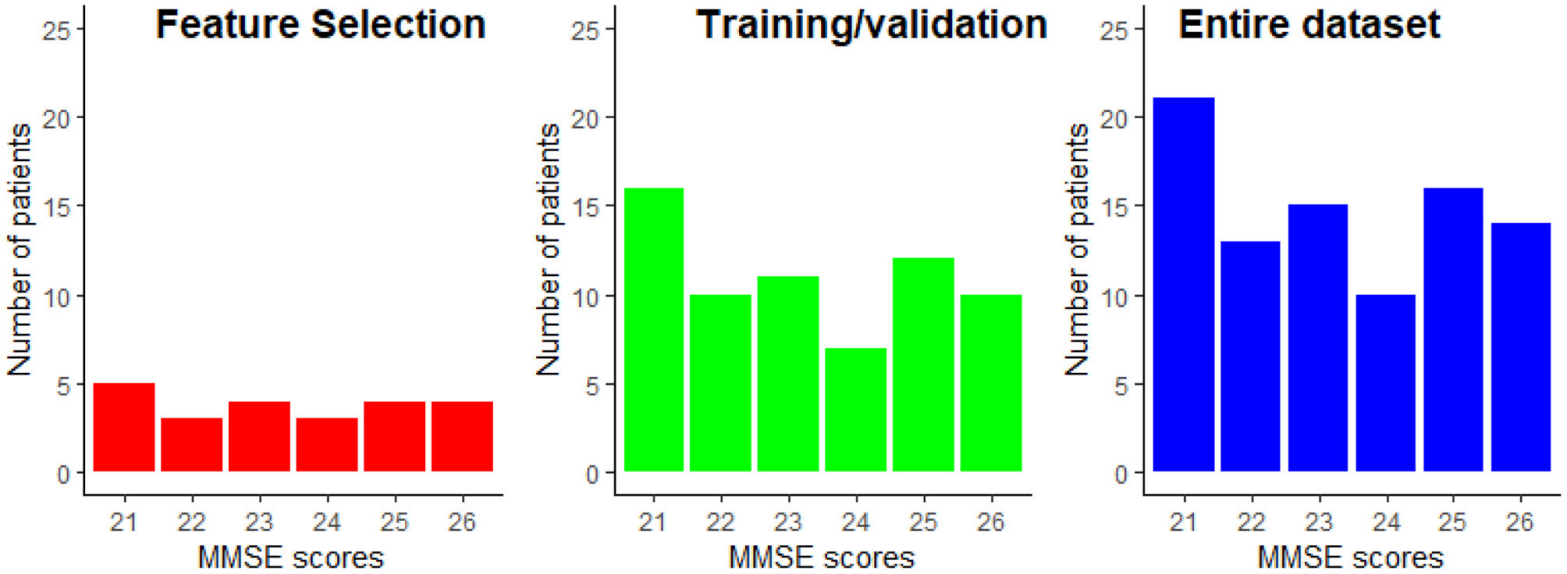
Example of the MMSE distributions for the feature selection and the train/validation datasets. The MMSE distribution of the entire dataset is provided as reference.

From the data available from the 89 patients, 25% were exclusively dedicated to the feature selection procedure, while the remaining 75% of the data was used for training and testing of the regression models. Using disjoint sets for feature selection and model training reduces any unwanted biases in the reported performance figures. Data partitioning was done randomly while keeping the distribution of the MMSE scores proportional to the entire dataset. The data partitioning is illustrated in Figure 5. This partitioning was done five times in order to assure that the selected features are not overly sensitive to data partitioning.

### 2.9. Regression Analysis

Since we are interested in AD severity monitoring, a regression task is needed. More specifically, we are interested in estimating the MMSE score of an AD patient. Motivated by insights from Cassani et al. (2018), three supervised regression models are explored (Gupta et al., 2019), namely: support vector machine regression with linear (SVM-Linear) and Gaussian (SVM-RBF) kernels, random forest regression (RF), and k-nearest neighbors regression (KNN). All the models were implemented with the open-source scikit-learn library for Python (Pedregosa et al., 2011). A brief summary of these regression models is detailed below for the sake of completeness.

#### 2.9.1. Support Vector Machine

The support vector machine regression (SVMR) model searches for a regression function in which all the obtained target errors will be under a specific value (Smola and Schölkopf, 2004). The strategy used in the implementation of the SVMR is very similar to the strategies used by the support vector machine classifier. In the regression scenario, the optimal hyperplane will not be used to separate the two sets of data but to map the training data into a regression function, and the margin in this scenario is used as a tolerance margin which the errors must be under (Rodríguez-Pérez et al., 2017).

#### 2.9.2. Random Forest

Random forest (RF), in turn, combines many decision trees into a single model. The structure of a random forest is composed of several decisions trees running in parallel with no interaction between them. Ultimately, the random forest outputs the mean value of each individual tree outputs that composes the random forest (Lebedev et al., 2014).

#### 2.9.3. k-Nearest Neighbors

The k-nearest neighbor classifier (KNN) assumes that an unseen sample likely belongs to the same class as the *k* most analogous-distant neighbors (Qin et al., 2013). For regression, the algorithm takes the weighted average of the *k* nearest neighbors, weighted by the inverse of their distance.

### 2.10. Evaluation Metrics

To evaluate each model's performance, we use the root mean squared error (RMSE), Pearson, and Spearman correlation between the predicted and the true MMSE score as metrics. Since the dataset available is not very large, we rely on repeated k-fold cross-validation as a testing setup. In this way, the available train/test dataset (i.e., 75% of the original dataset) is randomly split into k folds (per repetition). Training is done with k-1 folds and then tested on the held-out fold. This is repeated until all folds have been used as hold-out. With repeated cross-validation, this procedure is repeated *N* times, each time with a different partition. In the end, a total of *N* × *K* RMSE values are produced. Here, *N* = 50 and *K* = 5 are used. The average and standard deviation of the RMSE values across these *N* × *K* values are used to gauge system performance. In addition, average and standard deviation of Pearson and Spearman correlation calculated between predicted MMSE and observed MMSE per cross-validation value are also analyzed. Ideally, we are interested in models that i) achieve a low average RMSE, as well as a low standard deviation, thus suggesting the model is insensitive to data partitioning and ii) high correlation values.

## 3. Results and Discussion

### 3.1. Performance

In this section, we explore the benefits of AD severity prediction using unimodal and multimodal systems. Although we have investigated the performance of different feature selection and regression model algorithms, for the sake of brevity, the discussion will be based only on the results found from the utilization of MRMR for feature selection and random forest as a regression model, since they provided the best overall results. As an extension to the discussion, the interested reader is referred to the supplementary material where results from all the tested feature selection and regression model algorithms are presented.

**Figure 6:**
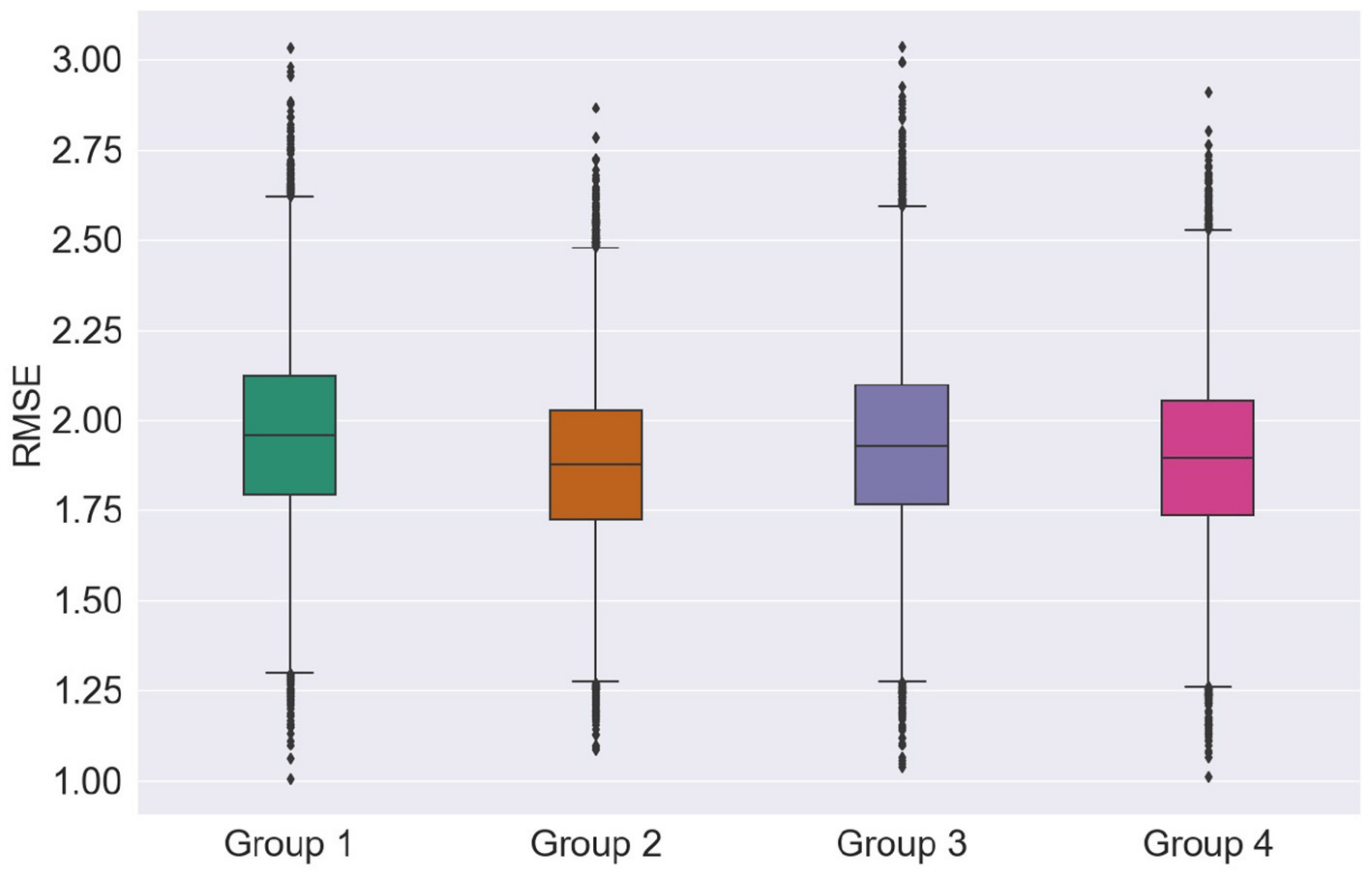
Distribution of the RMSE scores according to the groups of features.

First, we explore the effects of feature groups on regression accuracy. Here, we focus on the RMSE metric. Figure 6 presents the distributions of the overall average RMSE scores based on the different groups of features. If we look at the average and standard deviation of the RMSE scores computed over all the cross-validation trials and test runs, features from group 1 achieved 1.897 ± 0.231, features from group 2 of 1.928 ± 0.226, group 3 of 1.951 ± 0.238, and finally group 4 presented an average RMSE of 1.881 ± 0.228. For more details on the RMSE distributions per feature group for other regression models and feature selection algorithms, the reader is referred to Supplementary Table 1.

Looking at just average RMSE values, top EEG features seem to outperform the top MRI features in unimodal systems (group 1 and group 2), though not significantly. Figure 6, on the other hand, shows that MRI features achieved somewhat lower median, upper, and lower quartile RMSEs relative to EEG features, further corroborating recent findings by Farina et al. (2019). Overall, for most cases tested, multimodal systems, particularly trained on feature Group 4, showed slight improvements in performance relative to unimodal systems. In particular, lower RMSE variability was seen, thus suggesting that multimodal systems are less sensitive to data partitioning.

### 3.2. Feature Ranking

In order to better understand the impact of different feature modalities on overall severity monitoring performance, an in-depth analysis was performed to determine which features were consistently selected during the five test setup runs. Supplementary Tables 2–4 lists the features which were selected more than once by each of the feature selection techniques for Groups 1-3, respectively. The Tables demonstrate that MRMR is less sensitive to data partitioning than the other feature selection techniques, since it was able to select similar features across the five runs consistently. This was true for all feature groups tested. This is an important aspect that needs to also be taken into account when evaluating overall system performance (Khaire and Dhanalakshmi, in press).

The features *kurtosis* − *R*2*oR*3 − *P*3 − *P*4, *skewness* − *beta* − *mdelta* − *F*4, *cv* − *theta* − *mdelta* − *P*3, and *cv* − *R*2*oR*3 − *P*3 − *P*4 were shown to be those most often selected from the EEG modality, thus highlighting the importance of modulation spectral features not only for diagnostics, but also for AD severity monitoring. The virtual electrode *P*3 − *P*4 was also shown to be important, thus corroborating the inter-hemispheric deficit commonly reported with AD (Lakmache et al., 1998). More importantly, the recently-proposed modulation spectral patch features represented half of the top-20 EEG features selected by MRMR, thus highlighting their importance for unimodal EEG-based monitoring systems. From Supplementary Table 2, it can be seen that alpha and theta related features show up in the top rank, corroborating previous studies (Onishi et al., 2005), despite differences in the number of channels explored and the analysis protocol (eyes open vs. eyes closed) used.

From the MRI modality, in turn, a larger number of features derived from MRI scans were consistently selected (see Supplementary Table 3), relative to the EEG feature group. Area measures of the left frontal pole and superior temporal cortex were selected in all the runs by the MRMR technique, thus corroborating the importance of frontal and temporal lobes in the AD neuropathologic process (Kehoe et al., 2014). Furthermore, as severity increases, neurofibrillary tangles accumulate in greater numbers within the mediotemporal regions (including the entorhinal cortex), before extending beyond the limbic regions to involve neocortical association areas (Braak and Braak, 1991); as such, involvement of these frontal and temporal regions are expected.

Moreover, while AD is mostly associated with its effects on cortical gray matter, damage to white matter has also been reported. For example, differences have been noted in the scans of patients with AD compared to healthy controls in the white matter of the frontal, temporal, and parietal lobes (Bozzali et al., 2002; Salat et al., 2009). Here, MRMR selected gross measures of white matter volume: white matter mask volume, cerebral white matter volume, and estimated total intracranial volume, in four of the five runs. Right side measurements from the frontal pole, supramarginal, superior temporal, superior parietal, transverse temporal, cerebral white matter vol, unsegmented white matter, and insula were also selected in four out of the five runs, together with the left side measurement of cerebral white matter volume. Additionally, areas from the left and right side of lateral occipital, and white matter right-side measurements of the lateral occipital and fusiform were selected at least four times from Pearson and Spearman correlation feature selection techniques. These are all areas and features that have been previously linked to AD (e.g., Bottino et al., 2002; Fennema-Notestine et al., 2009; Leandrou et al., 2018; Farina et al., 2019). Encroachment of the neuropathology (e.g., neurofibrillary tangles) upon the occiptotemporal cortex has also been noted during the advancement of the disease (and has been linked to visuoperceptive and visuospatial deficits) (Braak and Braak, 1991).

Interestingly, of the 29 unique features consistently-selected from group 3 (see Supplementary Table 4), only six were from MRI. The top features also showed up previously during the unimodal tests, thus suggesting their importance for the task at hand.

Tables 4, 5 show the overall summary of consistently selected features based on feature category, frequency bands, electrode positions, and neuroanatomy. From the EEG features, the skewness features stood out in the unimodal systems, whereas the standard deviation features stood out for multimodal ones. Such findings suggest that feature temporal dynamics play an important role in severity monitoring; something that is corroborated by the importance seen with the modulation rate-of-change features. In fact, for both unimodal and multimodal systems, the amplitude modulation features were shown to be extremely important, in particular features related to the beta frequency subband dynamics, thus corroborating previous research (Jelles et al., 2008). The *R*2/*R*3 modulation patch regions, in turn, were extremely important for the unimodal systems.

**Table 4:** Distribution of the selected features that were selected in multiple runs for EEG groups.

**EEG**		
	**Unimodal**	**Multimodal**
**Summary statistics**		
Mean	2	4
Standard deviation	2	5
Coefficient of variation	3	4
Median	2	1
Skewness	21	4
Kurtosis	6	5
**Feature category**		
Spectral power	4	3
Coherence	5	5
Amplitude modulation	11	10
Modulation frequency	16	5
**Feature subband/region**		
Delta	2	1
Theta	3	-
Alpha	4	6
Beta	7	6
Gamma	4	5
R1	2	2
R3	1	-
R2/R3	10	2
R2/R1	3	1
**Electrode position**		
P3-P4	12	4
F4	3	-
P3	2	-
P4	3	2
F3-F4	6	3
F3	3	-
Pz	3	4
Cz	2	6
F3-P3	2	2
Fz	-	2

**Table 5:** Distribution of the selected features that were selected in multiple runs for MRI groups.

**MRI**		
	**Unimodal**	**Multimodal**
**Feature category**		
APARC	20	3
ASEG	7	1
WM	29	2
**Brain hemisphere**		
left	20	2
right	31	3

Regarding electrode positions, the virtual bipolar channels were important for unimodal systems, thus in line with previous research showing an inter-hemispheric disconnection with AD (Jeong, 2004). From the MRI features, in turn, features derived from the cortical parcellation and white matter volume ROIs were more often selected than subcortical volumetric features. Moreover, features from the right side of the brain showed up more often in the unimodal systems. Although some studies have shown greater left hemispheric deterioration with Alzheimer's disease (e.g., Karas et al., 2003; Thompson et al., 2003; Seo et al., 2007), our results may reflect that involvement of the right hemisphere (most likely in addition to early damage to the left) is associated with more widespread damage to cognitive systems and is therefore predictive of lower MMSE scores.

### 3.3. Models Based on Consistently-Selected Features

As a last experiment, we built models using only the consistently-selected features from the MRMR algorithm, as shown in Supplementary Tables 2–4 for EEG-only, MRI-only and EEG-MRI, respectively. Models are all based on the random forest regression model -and cross-validation testing scheme was used. In this experiment, the average RMSE obtained from the model with EEG features only was 1.798 ± 0.176, from the model with MRI features alone was 1.715 ± 0.186, and from the model with EEG-MRI features was 1.682 ± 0.177. As previously, for unimodal systems, the MRI-only model outperformed the EEG-only model, but multimodal systems were shown to always outperform at least one of the unimodal systems.

**Figure 7:**
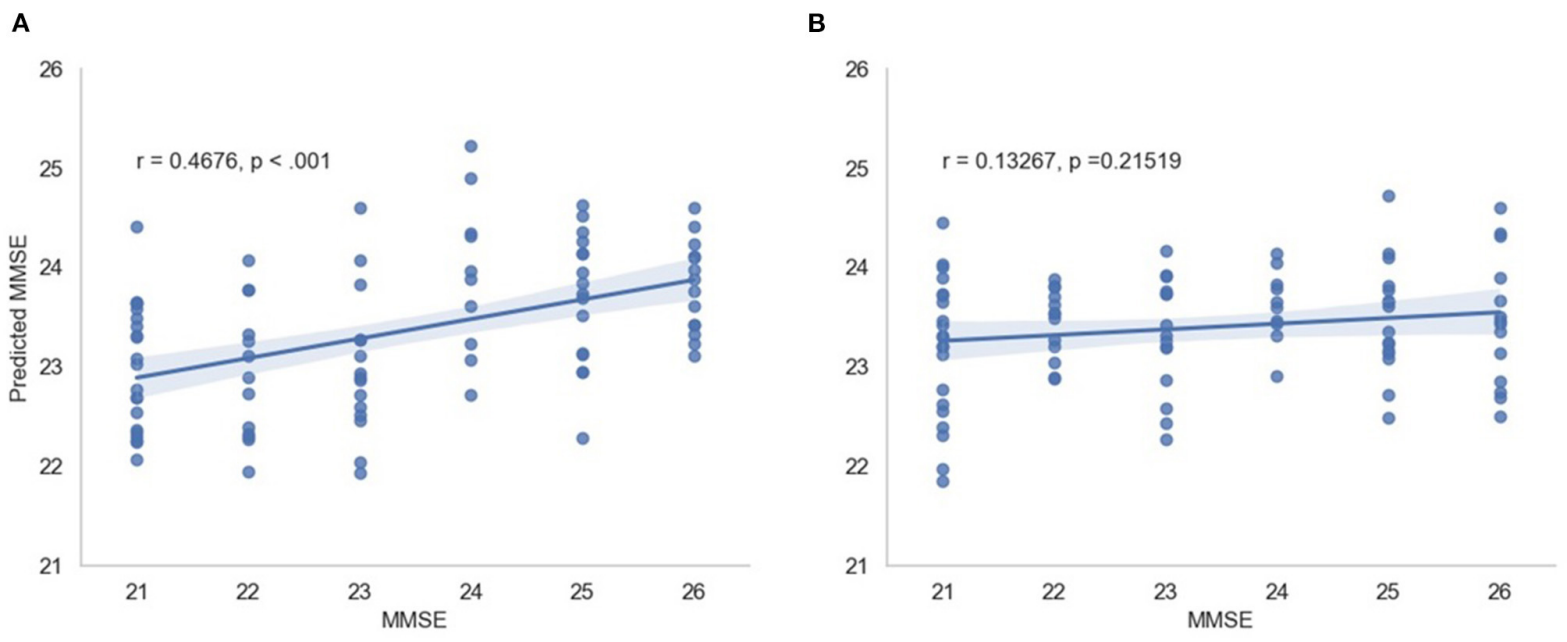
Predicted MMSE vs. observed MMSE for the cross-validation trial with the best performance for **(A)** the proposed model and **(B)** the random model.

In this scenario, we also report the Pearson and Spearman correlation coefficients calculated between the predicted MMSE and observed MMSE scores per cross-validation trial for the multimodal system. An average 0.350198 ± 0.049959 was attained for the Pearson correlation coefficient across all trials (all significant) and an average 0.348317 ± 0.052035 was obtained for the Spearman correlation coefficient (also significant). For visualization, Figure 7 depicts the predicted vs. observed MMSE scores for one of the cross-validation trials.

### 3.4. Statistical Significance of the Results

To test the significance of the obtained results, a “random regression model” was also developed. In this scenario, MMSE scores across patients were randomly permuted during the 50 x 5 cross-validation training phase of the regression models. RMSE scores of the random regression model were compared against the RMSE scores of the proposed models using a two-tail *t*-test. For all tested cases, the proposed method achieved lower RMSE values, suggesting better MMSE estimates. For all cases, with the exception of the SVM-RBF with group 1 and group 2 features, *p* < 0.025 were achieved, suggesting that the obtained errors were significantly lower than those achieved by chance. In addition, the “random regression model” for the scenario consisting of features from group 3 selected by the MMSE feature selection and a random forest regression model was randomly trained 500 times. All the random regression models' RMSE averages resulted in a higher value than the original RMSE of 1.68.

### 3.5. Study Limitations

The results reported in this study were performed on a limited sample size of 89 participants. While repeated cross-validation and multiple trial runs allowed for data partitioning biases to be explored, the generalization capability of the models to unseen patients is still unknown. Moreover, to the best of the authors' knowledge, no open-access dataset exists that includes EEG signals and MRI scans. The available data has a very narrow spread of MMSE scores, as shown in Figure 5. While this allows for models to be built for patients with minimal-mild levels of AD, it is still unknown how the models would behave as disease progresses into moderate and severe stages.

A further limitation is that a single marker of disease severity was used (i.e., MMSE), and it is unclear how the MRI/EEG features would perform when assessing severity using a different procedure. As such, further analyses are still needed. Moreover, model performances showed an improvement when features that presented stability across the feature selection experiment runs were used. In that regard, more recent feature selection techniques should be explored in the search and identification of more stable selected features, in particular using wrapper methods in which the feature selection and the regressor are tightly coupled. Given the importance seen of features extracted from structural MRI, it could be the case that other measures derived from functional MRI, such as functional connectivity measures, could show complementary information for multimodal systems.

In this study, a range of neuroanatomical measures were provided directly via the outputs from FreeSurfer, but additional or alternative measures could be incorporated in the future, for example, by including neuroanatomical measures (e.g., cortical volume ROIs) corrected for head size (i.e., by using estimated total intracranial volume), measures of hippocampal subfields, laterality indices, or structural covariance, to name a few. As the MRI data utilized in this study were collected in different sites with different protocols, further studies should explore the effects of different normalization procedures relative to scanner sites and/or field strengths. While the available data was not sufficient for such an analysis, a preliminary exploration was conducted and we compared the initial model trained on all data (*n* = 89 participants) to a model trained from data from the single largest site (*n* = 43 participants). Using a two-tail t-test to compare the estimates from both models, we found that for group 3 and group 4 feature sets, *p* = 0.15 and *p* = 0.67 were achieved, respectively. This suggests that the results are not significantly different to each other and that additional normalization may not be crucial. Notwithstanding, further studies with a larger population sizes are needed to obtain more conclusive results.

Lastly, with the advances seen with deep neural network based analyses of EEG (Roy et al., 2019) and MRI signals (Jo et al., 2019), deep multimodal architectures should be explored once more data is made available to the research community.

## 4. Conclusion

Although features such as beta-amyloid or tau may be used to illustrate the progression of the hallmark neuropathology relating to Alzheimer's disease, additional combinations of neural features captured through EEG and structural MRI may provide further insight on the neurodegeneration and neuronal injury (including synaptic dysfunction) that occurs with disease progression. This paper has explored the use of both structural MRI and EEG features, typically developed for Alzheimer's disease diagnosis, for the purpose of monitoring disease severity (as measured by the MMSE). Various feature selection algorithms were tested, along with cross-modality fusion schemes, and machine learning algorithms. Experimental results showed that while new EEG modulation spectral patch features are more relevant than conventional spectral power ones for AD severity level monitoring, systems trained solely on MRI features tended to outperform EEG ones. Overall, multimodal EEG-MRI systems showed improved accuracy and lower variability across cross-validation runs, thus suggesting their importance for practical applications relating to the monitoring of disease progression. In relation to the feature selection algorithms, the MRMR algorithm showed improved stability across different test setup runs and stood out among the other correlation-based candidates. Across machine learning algorithms, non-linear regression models showed improved accuracy over linear ones.

## Data Availability Statement

The raw data supporting the conclusions of this article will be made available by the authors, without undue reservation. Requests to access these datasets should be directed to Marco Cecchi (mcecchi@cognision.com).

## Ethics Statement

Ethical review and approval was not required for the study on human participants in accordance with the local legislation and institutional requirements. The patients/participants provided their written informed consent to participate in this study.

## Author Contributions

BJ, RC, WM, and TF contributed to the conception and design of the study and determined the features utilized in the study. MC and KF collected and organized the database. BJ performed the analyses presented in the paper. BJ, WM, and TF wrote the first draft of the manuscript and all authors contributed to manuscript revision, and approval of the submitted version.

## Author Disclaimer

The work presented in this paper has been conducted and presented by the first author during his master thesis research. The thesis has already been defended and shows up publicly at the institution's website. However, the authors would like to declare that this work is original and has not been published or submitted to any other journals.

## Conflict of Interest

MC and KF were employed by the company COGNISION. The remaining authors declare that the research was conducted in the absence of any commercial or financial relationships that could be construed as a potential conflict of interest.

## Publisher's Note

All claims expressed in this article are solely those of the authors and do not necessarily represent those of their affiliated organizations, or those of the publisher, the editors and the reviewers. Any product that may be evaluated in this article, or claim that may be made by its manufacturer, is not guaranteed or endorsed by the publisher.

## Footnotes

^1^https://surfer.nmr.mgh.harvard.edu.

## References

[B1] Alzheimer's Association (2019). 2019 Alzheimer's disease facts and figures. Alzheimers Dement. 15, 321–387. 10.1016/j.jalz.2019.01.010

[B2] Bagherzadeh-Khiabani F. Ramezankhani A. Azizi F. Hadaegh F. Steyerberg E. W. Khalili D. (2016). A tutorial on variable selection for clinical prediction models: feature selection methods in data mining could improve the results. J. Clin. Epidemiol. 71, 76–85. 10.1016/j.jclinepi.2015.10.002 26475568

[B3] Bottino C. M. Castro C. C. Gomes R. L. Buchpiguel C. A. Marchetti R. L. Neto M. R. L. (2002). Volumetric mri measurements can differentiate Alzheimer's disease, mild cognitive impairment, and normal aging. Int. Psychogeriatr. 14, 59–72. 10.1017/S1041610202008281 12094908

[B4] Bozzali M. Falini A. Franceschi M. Cercignani M. Zuffi M. Scotti G.. (2002). White matter damage in Alzheimer's disease assessed *in vivo* using diffusion tensor magnetic resonance imaging. J. Neurol. Neurosurg. Psychiatry 72, 742–746. 10.1136/jnnp.72.6.742 12023417 PMC1737921

[B5] Braak H. Braak E. (1991). Neuropathological stageing of Alzheimer-related changes. Acta Neuropathol. 82, 239–259. 10.1007/BF00308809 1759558

[B6] Cai J. Luo J. Wang S. Yang S. (2018). Feature selection in machine learning: a new perspective. Neurocomputing 300:70–79. 10.1016/j.neucom.2017.11.077

[B7] Cardinale F. Giuseppa C. Bramerio M. Mai R. Sartori I. Cossu M.. (2014). Validation of freesurfer-estimated brain cortical thickness: Comparison with histologic measurements. Neuroinformatics 12, 535–542. 10.1007/s12021-014-9229-2 24789776

[B8] Caroli A. Frisoni G. Initiative A. D. N.. (2010). The dynamics of Alzheimer's disease biomarkers in the Alzheimer's disease neuroimaging initiative cohort. Neurobiol. Aging. 31, 1263–1274. 10.1016/j.neurobiolaging.2010.04.024 20538373 PMC3467365

[B9] Casey D. A. (2010). Event-related potentials and the diagnosis of Alzheimer's disease—the cognision™ system. US Neurol. 6, 34–36. 10.17925/USN.2010.06.02.34

[B10] Cassani R. Estarellas M. San-Martin R. Fraga F. J. Falk T. H. (2018). Systematic review on resting-state eeg for Alzheimer's disease diagnosis and progression assessment. Dis. Markers 2018:5174815. 10.1155/2018/5174815 30405860 PMC6200063

[B11] Cassani R. Falk T. (2020). Alzheimer's disease diagnosis and severity level detection based on electroencephalography modulation spectral “patch” features. IEEE J. Biomed. Health Inform. 24, 1982–1993. 10.1109/JBHI.2019.2953475 31725401

[B12] Cassani R. Falk T. H. Fraga F. J. Cecchi M. Moore D. K. Anghinah R. (2017). Towards automated electroencephalography-based Alzheimer's disease diagnosis using portable low-density devices. Biomed. Signal Process Control. 33, 261–271. 10.1016/j.bspc.2016.12.009

[B13] Cassani R. Falk T. H. Fraga F. J. Kanda P. A. Anghinah R. (2014a). The effects of automated artifact removal algorithms on electroencephalography-based Alzheimer's disease diagnosis. Front. Aging Neurosci. 6:55. 10.3389/fnagi.2014.00055 24723886 PMC3971195

[B14] Cassani R. Falk T. H. Fraga F. J. Kanda P. A. Anghinah R. (2014b). Towards automated eeg-based Alzheimer's disease diagnosis using relevance vector machines, in 5th ISSNIP-IEEE Biosignals and Biorobotics Conference, Biosignals and Robotics for Better and Safer Living (BRC) (Salvador: IEEE), 1–6.

[B15] Cecchi M. Moore D. K. Sadowsky C. H. Solomon P. R. Doraiswamy P. M. Smith C. D.. (2015). A clinical trial to validate event-related potential markers of Alzheimer's disease in outpatient settings. Alzheimers Dement. 1, 387–394. 10.1016/j.dadm.2015.08.004 27239520 PMC4879492

[B16] Chetelat G. Baron J.-C. (2003). Early diagnosis of Alzheimer's disease: contribution of structural neuroimaging. Neuroimage 18, 525–541. 10.1016/S1053-8119(02)00026-5 12595205

[B17] Chok N. S. (2010). Pearson's Versus Spearman's and Kendall's Correlation Coefficients for Continuous Data (Ph.D. thesis). University of Pittsburgh.

[B18] Dale A. M. Fischl B. Sereno M. I. (1999). Cortical surface-based analysis. i. segmentation and surface reconstruction. Neuroimage 9, 179–194. 10.1006/nimg.1998.0395 9931268

[B19] Desikan R. S. Ségonne F. Fischl B. Quinn B. T. Dickerson B. C. Blacker D.. (2006). An automated labeling system for subdividing the human cerebral cortex on mri scans into gyral based regions of interest. Neuroimage 31, 968–980. 10.1016/j.neuroimage.2006.01.021 16530430

[B20] Devore J. L. (2008). Probability and Statistics for Engineering and the Sciences. Boston, MA: Spinger.

[B21] Dormann C. F. Elith J. Bacher S. Buchmann C. Carl G. Carré G.. (2013). Collinearity: a review of methods to deal with it and a simulation study evaluating their performance. Ecography 36, 27–46. 10.1111/j.1600-0587.2012.07348.x

[B22] Eskildsen S. F. Coupé P. Fonov V. S. Pruessner J. C. Collins D. L. Initiative A. D. N.. (2015). Structural imaging biomarkers of Alzheimer's disease: predicting disease progression. Neurobiol. Aging 36, S23–S31. 10.1016/j.neurobiolaging.2014.04.034 25260851

[B23] Falahati F. Westman E. Simmons A. (2014). Multivariate data analysis and machine learning in Alzheimer's disease with a focus on structural magnetic resonance imaging. J. Alzheimers Dis. 41, 685–708. 10.3233/JAD-131928 24718104

[B24] Falk T. H. Fraga F. J. Trambaiolli L. Anghinah R. (2012). Eeg amplitude modulation analysis for semi-automated diagnosis of Alzheimer's disease. EURASIP J. Adv. Signal Process 2012, 192. 10.1186/1687-6180-2012-192

[B25] Farina F. Emek-Savaş D. Rueda-Delgado L. Boyle R. Kiiski H. Yener G.. (2019). A comparison of resting state eeg and structural mri for classifying Alzheimer's disease and mild cognitive impairment. bioRxiv. 10.1101/711465 32278090

[B26] Fennema-Notestine C. Hagler D. J. Jr McEvoy L. K. Fleisher A. S. Wu E. H. Karow D. S.. (2009). Structural mri biomarkers for preclinical and mild Alzheimer's disease. Hum. Brain Mapp. 30, 3238–3253. 10.1002/hbm.20744 19277975 PMC2951116

[B27] Fischl B. (2012). Freesurfer. Neuroimage 62, 774–781. 10.1016/j.neuroimage.2012.01.021 22248573 PMC3685476

[B28] Fischl B. Dale A. M. (2000). Measuring the thickness of the human cerebral cortex from magnetic resonance images. Proc. Natl. Acad. U.S.A. 97, 11050–11055. 10.1073/pnas.200033797 10984517 PMC27146

[B29] Fischl B. Salat D. H. Busa E. Albert M. Dieterich M. Haselgrove C.. (2002). Whole brain segmentation: automated labeling of neuroanatomical structures in the human brain. Neuron 33, 341–355. 10.1016/S0896-6273(02)00569-X 11832223

[B30] Fischl B. Sereno M. I. M. D. A. (1999). Cortical surface-based analysis. ii. inflation, flattening, and a surface-based coordinate system. Neuroimage 9, 195–207. 10.1006/nimg.1998.0396 9931269

[B31] Fischl B. Van Der Kouwe A. Destrieux C. Halgren E. Ségonne F. Salat D. H.. (2004). Automatically parcellating the human cerebral cortex. Cereb. Cortex 14, 11–22. 10.1093/cercor/bhg087 14654453

[B32] Folstein M. F. Folstein S. E. McHugh P. R. (1975). mini-mental state”: a practical method for grading the cognitive state of patients for the clinician. J. Psychiatr. Res. 12, 189–198. 10.1016/0022-3956(75)90026-6 1202204

[B33] Fraga F. J. Falk T. H. Trambaiolli L. R. Oliveira E. F. Pinaya W. H. Kanda P. A.. (2013). Towards an eeg-based biomarker for Alzheimer's disease: Improving amplitude modulation analysis features, in 2013 IEEE International Conference on Acoustics, Speech and Signal Processing (Vancouver, BC: IEEE), 1207–1211.

[B34] Freidl W. Schmidt R. Stronegger W. J. Irmler A. Reinhart B. Koch M. (1996). Mini mental state examination: influence of sociodemographic, environmental and behavioral factors and vascular risk factors. J. Clin. Epidemiol. 49, 73–78. 10.1016/0895-4356(95)00541-2 8598514

[B35] Frisoni G. B. Fox N. C. Jack C. R. Scheltens P. Thompson P. M. (2010). The clinical use of structural mri in Alzheimer disease. Nat. Rev. Neurol. 6, 67–77. 10.1038/nrneurol.2009.215 20139996 PMC2938772

[B36] Galasko D. Abramson I. Corey-Bloom J. Thal L. J. (1993). Repeated exposure to the mini-mental state examination and the information-memory-concentration test results in a practice effect in Alzheimer's disease. Neurology 43, 1559–1563. 10.1212/WNL.43.8.1559 8351011

[B37] Gross A. L. Mungas D. M. Leoutsakos J.-M. S. Albert M. S. Jones R. N. Initiative A. D. N.. (2016). Alzheimer's disease severity, objectively determined and measured. Alzheimers Dement. 4, 159–168. 10.1016/j.dadm.2016.08.005 27830173 PMC5078784

[B38] Gupta Y. Lee K. H. Choi K. Y. Lee J. J. Kim B. C. Kwon G. R.. (2019). Early diagnosis of Alzheimer's disease using combined features from voxel-based morphometry and cortical, subcortical, and hippocampus regions of mri t1 brain images. PLoS ONE 14:e0222446. 10.1371/journal.pone.0222446 31584953 PMC6777799

[B39] Hampel H. Frank R. Broich K. Teipel S. J. Katz R. G. Hardy J.. (2010). Biomarkers for Alzheimer's disease: academic, industry and regulatory perspectives. Nat. Rev. Drug Discov. 9, 560–574. 10.1038/nrd3115 20592748

[B40] Jack C. R. Bennett D. A. Blennow K. Carrillo M. C. Feldman H. H. Frisoni G. B.. (2016). A/t/n: an unbiased descriptive classification scheme for Alzheimer disease biomarkers. Neurology 87, 539–547. 10.1212/WNL.0000000000002923 27371494 PMC4970664

[B41] Jedynak B. M. Lang A. Liu B. Katz E. Zhang Y. Wyman B. T.. (2012). A computational neurodegenerative disease progression score: method and results with the Alzheimer's disease neuroimaging initiative cohort. Neuroimage 63, 1478–1486. 10.1016/j.neuroimage.2012.07.059 22885136 PMC3472161

[B42] Jelles B. Scheltens P. Van der Flier W. Jonkman E. da Silva F. L. Stam C. (2008). Global dynamical analysis of the eeg in Alzheimer's disease: frequency-specific changes of functional interactions. Clin. Neurophysiol. 119, 837–841. 10.1016/j.clinph.2007.12.002 18258479

[B43] Jeong J. (2004). Eeg dynamics in patients with Alzheimer's disease. Clin. Neurophysiol. 115, 1490–1505. 10.1016/j.clinph.2004.01.001 15203050

[B44] Jo T. Nho K. Saykin A. J. (2019). Deep learning in Alzheimer's disease: diagnostic classification and prognostic prediction using neuroimaging data. Front Aging Neurosci. 11:220. 10.3389/fnagi.2019.00220 31481890 PMC6710444

[B45] Jović A. Brkić K. Bogunović N. (2015). A review of feature selection methods with applications, in 2015 38th International Convention on Information and Communication Technology, Electronics and Microelectronics (MIPRO) (Opatija: IEEE), 1200–1205.

[B46] Karas G. Burton E. Rombouts S. van Schijndel R. O'Brien J. Scheltens P.. (2003). A comprehensive study of gray matter loss in patients with Alzheimer's disease using optimized voxel-based morphometry. Neuroimage 18, 895–907. 10.1016/S1053-8119(03)00041-7 12725765

[B47] Kehoe E. G. McNulty J. P. Mullins P. G. Bokde A. L. (2014). Advances in mri biomarkers for the diagnosis of Alzheimer's disease. Biomark Med. 8, 1151–1169. 10.2217/bmm.14.42 25402585

[B48] Khaire U. M. Dhanalakshmi R. (in press). Stability of feature selection algorithm: a review. J. King Saud Univer. Comput. Inf. Sci. 10.1016/j.jksuci.2019.06.012

[B49] Kowalski J. W. Gawel M. Pfeffer A. Barcikowska M. (2001). The diagnostic value of eeg in Alzheimer disease: correlation with the severity of mental impairment. J. Clin. Neurophysiol. 18, 570–575. 10.1097/00004691-200111000-00008 11779971

[B50] Kuskowski M. A. Mortimer J. A. Morley G. K. Malone S. M. Okaya A. J. (1993). Rate of cognitive decline in Alzheimer's disease is associated with eeg alpha power. Biol. Psychiatry 33, 659–662. 10.1016/0006-3223(93)90108-P 8329497

[B51] Lakmache Y. Lassonde M. Gauthier S. Frigon J.-Y. Lepore F. (1998). Interhemispheric disconnection syndrome in Alzheimer's disease. Proc. Natl. Acad. Sci. U.S.A. 95, 9042–9046. 10.1073/pnas.95.15.9042 9671801 PMC21199

[B52] Leandrou S. Petroudi S. Kyriacou P. A. Reyes-Aldasoro C. C. Pattichis C. S. (2018). Quantitative mri brain studies in mild cognitive impairment and A l lzheimer's disease: a methodological review. IEEE Rev. Biomed. Eng. 11, 97–111. 10.1109/RBME.2018.2796598 29994606

[B53] Lebedev A. Westman E. Van Westen G. Kramberger M. Lundervold A. Aarsland D.. (2014). Random forest ensembles for detection and prediction of alzheimer's disease with a good between-cohort robustness. Neuroimage Clin. 6, 115–125. 10.1016/j.nicl.2014.08.023 25379423 PMC4215532

[B54] Li J. Cheng K. Wang S. Morstatter F. Trevino R. P. Tang J.. (2017). Feature selection: a data perspective. ACM Comput. Surv. 50, 1–45. 10.1145/3136625

[B55] Mammone N. La Foresta F. Morabito F. C. (2011). Automatic artifact rejection from multichannel scalp eeg by wavelet ica. IEEE Sens J. 12, 533–542. 10.1109/JSEN.2011.2115236

[B56] Martínez-Torteya A. Treviño V. Tamez-Peña J. G. (2015). Improved diagnostic multimodal biomarkers for alzheimer's disease and mild cognitive impairment. Biomed. Res. Int. 2015:961314. 10.1155/2015/961314 26106620 PMC4464003

[B57] Mayeux R. Schupf N. (2011). Blood-based biomarkers for alzheimer's disease: plasma aβ40 and aβ42, and genetic variants. Neurobiol. Aging 32, S10–S19. 10.1016/j.neurobiolaging.2011.09.004 22078169 PMC3233700

[B58] McGeown W. J. Cassani R. Falk T. H. Cecchi M. Fadem K. (2019). P2-364: Neuroanatomical and neuropsychological correlates of resting state eeg diagnostic features in patients with Alzheimer's disease. Alzheimers Dement. 15(7S_Part_14):P739–P740. 10.1016/j.jalz.2019.06.2771

[B59] Nasreddine Z. S. Phillips N. A. Bédirian V. Charbonneau S. Whitehead V. Collin I.. (2005). The montreal cognitive assessment, moca: a brief screening tool for mild cognitive impairment. J. Am. Geriatr. Soc. 53, 695–699. 10.1111/j.1532-5415.2005.53221.x 15817019

[B60] Onishi J. Suzuki Y. Yoshiko K. Hibino S. Iguchi A. (2005). Predictive model for assessing cognitive impairment by quantitative electroencephalography. Cogn. Behav. Neurol. 18, 179–184. 10.1097/01.wnn.0000178227.54315.38 16175023

[B61] Pedregosa F. Varoquaux G. Gramfort A. Michel V. Thirion B. Grisel O.. (2011). Scikit-learn: machine learning in python. J. Mach. Learn. Res. 12, 2825–2830. Available online at: https://www.jmlr.org/papers/v12/pedregosa11a.html

[B62] Peng H. Long F. Ding C. (2005). Feature selection based on mutual information: criteria of max-dependency, max-relevance, and min-redundancy. IEEE Trans. Pattern Anal. Mach. Intell. 27, 1226–1238. 10.1109/TPAMI.2005.159 16119262

[B63] Qin Z. Wang A. T. Zhang C. Zhang S. (2013). Cost-sensitive classification with k-nearest neighbors, in International Conference on Knowledge Science, Engineering and Management (Berlin; Heidelberg: Springer), 112–131.

[B64] Qiu R. G. Qiu J. L. Badr Y. (2017). Predictive modeling of the severity/progression of alzheimer's diseases, in 2017 International Conference on Grey Systems and Intelligent Services (GSIS) (Stockholm,: IEEE), 400–403.

[B65] Ramos A. R. Dong C. Elkind M. S. Boden-Albala B. Sacco R. L. Rundek T.. (2013). Association between sleep duration and the mini-mental score: the Northern Manhattan study. J. Clin. Sleep. Med. 9, 669–673. 10.5664/jcsm.2834 23853560 PMC3671331

[B66] Rodríguez-Pérez R. Vogt M. Bajorath J. (2017). Support vector machine classification and regression prioritize different structural features for binary compound activity and potency value prediction. ACS Omega 2, 6371–6379. 10.1021/acsomega.7b01079 30023518 PMC6045367

[B67] Roy Y. Banville H. Albuquerque I. Gramfort A. Falk T. H. Faubert J. (2019). Deep learning-based electroencephalography analysis: a systematic review. J. Neural Eng. 16, 051001. 10.1088/1741-2552/ab260c 31151119

[B68] Salat D. H. Greve D. N. Pacheco J. L. Quinn B. T. Helmer K. G. Buckner R. L.. (2009). Regional white matter volume differences in nondemented aging and alzheimer's disease. Neuroimage 44, 1247–1258. 10.1016/j.neuroimage.2008.10.030 19027860 PMC2810540

[B69] Seo S. W. Im K. Lee J.-M. Kim Y.-H. Kim S. T. Kim S. Y.. (2007). Cortical thickness in single- versus multiple-domain amnestic mild cognitive impairment. Neuroimage 36:289–297. 10.1016/j.neuroimage.2007.02.042 17459730

[B70] Smola A. J. Schölkopf B. (2004). A tutorial on support vector regression. Stat Comput. 14, 199–222. 10.1023/B:STCO.0000035301.49549.88

[B71] Thompson P. M. Hayashi K. M. De Zubicaray G. Janke A. L. Rose S. E. Semple J.. (2003). Dynamics of gray matter loss in alzheimer's disease. J. Neurosci. 23, 994–1005. 10.1523/JNEUROSCI.23-03-00994.2003 12574429 PMC6741905

[B72] Van Cauwenberghe C. Van Broeckhoven C. Sleegers K. (2016). The genetic landscape of alzheimer disease: clinical implications and perspectives. Genet. Med. 18, 421–430. 10.1038/gim.2015.117 26312828 PMC4857183

[B73] Wild K. Howieson D. Webbe F. Seelye A. Kaye J. (2008). Status of computerized cognitive testing in aging: a systematic review. Alzheimers Dement. 4, 428–437. 10.1016/j.jalz.2008.07.003 19012868 PMC2645803

[B74] Zhang D. Wang Y. Zhou L. Yuan H. Shen D. Initiative A. D. N.. (2011). Multimodal classification of alzheimer's disease and mild cognitive impairment. Neuroimage 55, 856–867. 10.1016/j.neuroimage.2011.01.008 21236349 PMC3057360

