# Supplementary material for: Multimodal Prediction of Alzheimer's Disease Severity Level Based on Resting-State EEG and Structural MRI
Source: Front Hum Neurosci. 2021 Sep 9;15:700627. doi: 10.3389/fnhum.2021.700627 (PMC8458963; doi:10.3389/fnhum.2021.700627)
Supplement: Supplementary file 1 [file Data_Sheet_1.pdf]

Supplementary Material

1 SUPPLEMENTARY DATA

1.1 Subcortical Segmentation

Volumes of anatomical structures included: lateral ventricle, inferior lateral ventricle, cerebellum white matter, cerebellum cortex, thalamus proper, caudate, putamen, pallidum, hippocampus, amygdala, accumbens area, ventral DC, vessel, choroid plexus, cortex, cerebral white matter, and surface holes were determined for the left and right hemispheres. The 3rd ventricle, 4th ventricle, 5th ventricle, cerebrospinal fluid (CSF), WM hypointensities, non WM hypointensities, optic chiasm, posterior corpus callosum, midposterior corpus callosum, central corpus callosum, midanterior corpus callosum, anterior corpus callosum, brain segmentation, brain segmentation without ventricles, brain segmentation without ventricles from surface reconstruction, cortex, cerebral white matter, subcortical gray matter, total gray matter, supratentorial, supratentorial without ventricles, supratentorial without ventricles (voxel-based), mask, ratio of brain segmentation to estimated total intracranial volume (eTIV), ratio of mask to eTIV, total number of defect holes in surfaces before fixing, and estimated total intracranial volume features were also measured. The features WM hypointensities and non WM hypointensities were not considered in the analysis because they presented no variance among the subjects.

1.2 Cortical Parcellation

Bankssts (cortical areas around superior temporal sulcus), caudal anterior cingulate, caudal middle frontal, cuneus, entorhinal, fusiform, inferior parietal, inferior temporal, isthmus cingulate, lateral occipital, lateral orbitofrontal, lingual, medial orbitofrontal, middle temporal, parahippocampal, paracentral, pars opercularis, pars orbitalis, pars triangularis, pericalcarine, postcentral, posterior cingulate, precentral, precuneus, rostral anterior cingulate, rostral middle frontal, superior frontal, superior parietal, superior temporal, supramarginal, frontal pole, temporal pole, transverse temporal and insula. Additional outputs included mean thickness, thickness from brain segmentation volume without ventricles, thickness from eTIV, white surface area, area from brain segmentation volume without ventricles, and area from eTIV.

1.3 White Matter Parcellation

Bankssts, caudal anterior cingulate, caudal middle frontal, cuneus, entorhinal, fusiform, inferior parietal, inferior temporal, isthmus cingulate, lateral occipital, lateral orbitofrontal, lingual, medial orbitofrontal, middle temporal, parahippocampal, paracentral, pars opercularis, pars orbitalis, pars triangularis, pericalcarine, postcentral, posterior cingulate, precentral, precuneus, rostral anterior cingulate, rostral middlefrontal, superior frontal, superior parietal, superior temporal, supramarginal, frontal pole, temporal pole, transverse temporal, insula, unsegmented white matter, and cerebral white matter. Furthermore, the entire cerebral white matter volume, mask volume, and estimated total intracranial volume were determined.

2 SUPPLEMENTARY TABLES AND FIGURES

Experimental results for the four feature groups are shown in Table S1, where the average RMSE score followed by its standard deviation is reported over all the cross-validation trials and averaged over the five test setup runs. Moreover, Figures S1 and S2 present the distributions of the overall average RMSE scores based on the different regression algorithms, different groups of features, and the different feature selection algorithms, respectively.

Table S1. Performance comparison across regressors, feature selection algorithms, and feature groups.

MRMR	Group 1	Group 2	Group 3	Group 4
SVM Linear	2.065 ± 0.269	1.847 ± 0.208	2.018 ± 0.245	1.931 ± 0.244
SVM RBF	1.849 ± 0.198	1.833 ± 0.196	1.845 ± 0.198	**1.830 ± 0.194**
Random Forest	1.897 ± 0.231	1.928 ± 0.226	1.951 ± 0.238	1.881 ± 0.228
KNN	2.009 ± 0.261	1.894 ± 0.242	2.017 ± 0.276	2.011 ± 0.265

Pearson	Group 1	Group 2	Group 3	Group 4
SVM Linear	2.021 ± 0.261	1.859 ± 0.218	1.958 ± 0.259	1.924 ± 0.246
SVM RBF	1.843 ± 0.195	**1.828 ± 0.195**	1.835 ± 0.194	1.834 ± 0.194
Random Forest	1.963 ± 0.231	1.844 ± 0.247	1.889 ± 0.257	1.840 ± 0.245
KNN	2.057 ± 0.256	1.971 ± 0.258	2.010 ± 0.272	1.949 ± 0.248

Spearman	Group 1	Group 2	Group 3	Group 4
SVM Linear	2.019 ± 0.255	1.864 ± 0.209	1.968 ± 0.257	1.920 ± 0.245
SVM RBF	1.848 ± 0.194	1.830 ± 0.195	1.841 ± 0.193	1.834 ± 0.193
Random Forest	1.917 ± 0.223	1.849 ± 0.245	1.854 ± 0.238	**1.827 ± 0.240**
KNN	2.031 ± 0.253	1.952 ± 0.251	2.009 ± 0.265	1.954 ± 0.257

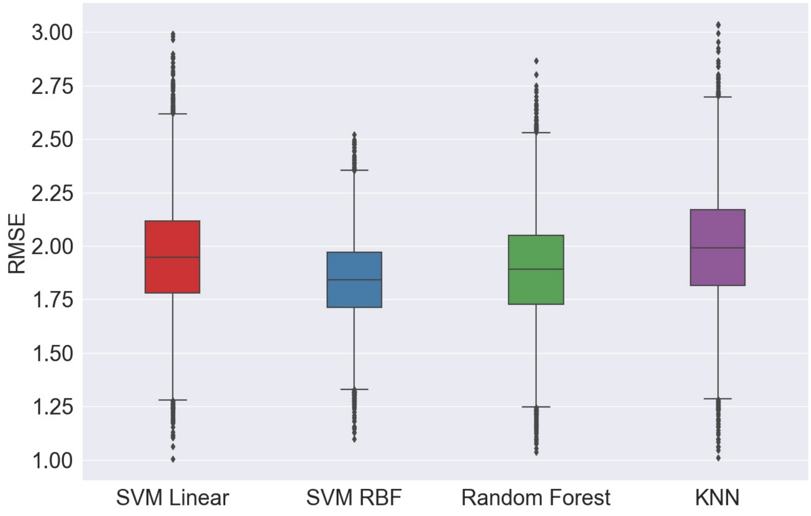

Figure S1. Distribution of the RMSE scores according to the models.

As can be seen from Table S1 and Figure S1, the results achieved suggest that SVM-RBF and random forest algorithms achieve lower prediction errors relative to an SVM with a linear kernel or a kNN regressor. The SVM-RBF regressor, in turn, achieved a lower RMSE variability, suggesting the model is less sensitive to data partitioning, an important factor for system generality. Moreover, the results from Table S1 and Figure S2 suggest that feature selection algorithms do not play a crucial role on final performance. While a random forest regressor with Group 4 features resulted in the lower average RMSE (1.827 ± 0.240), the variability was rather large. For practical applications, however, a combination of lower RMSE average and variability is desirable. As such, the SVM-RBF method, combined with either Group 3 (1.845 ± 0.198,

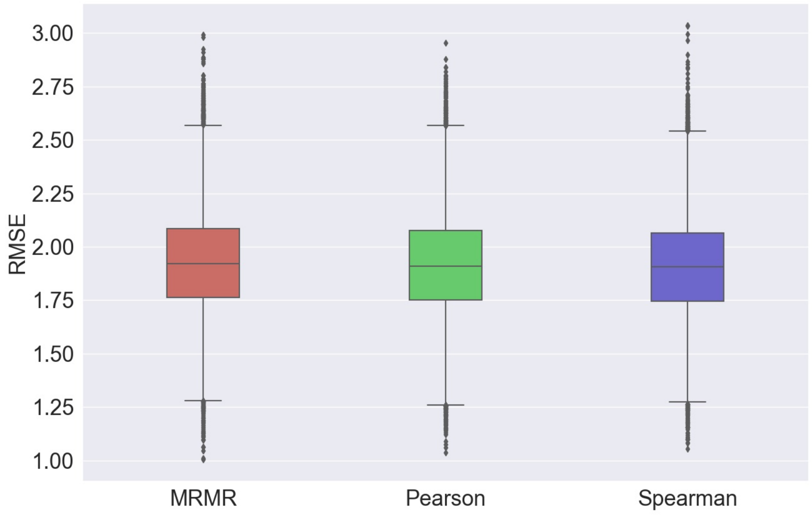

Figure S2. Distribution of the RMSE scores according to the features selection techniques.

1.835 ± 0.194, 1.841 ± 0.193) or Group 4 (1.830 ± 0.194, 1.834 ± 0.194, 1.834 ± 0.193) features, showed to be the most promising solutions from purely a performance point of view.

Tables S2, S3, and S4 show the list of the features which were selected more than once by each of the feature selection techniques for feature Groups 1-3, respectively.

As a last experiment, we built models using only the consistently-selected features from the MRMR algorithm, as shown in supplementary material Tables for EEG-only, MRI-only and EEG-MRI, respectively. Table S5 presents the results obtained from the four regression algorithms in a cross-validation testing scheme. The SMV-RBF achieved the lowest variability across the cross-validation trials. As previously, the SVM-RBF and random forest methods achieved the best performance, thus further hinting at the importance of non-linear models.

Table S2. Consistently Selected Features from Group 1 - EEG Features.

**Runs**	**Elements**
	**MRMR**
1, 2, 3, 4, and 5	kurtosis-R2oR3-P3-P4
1, 2, 3, and 5	cv-theta-mdelta-P3
1, 3, 4, and 5	skewness-beta-mdelta-F4
1, 2, and 3	cv-R2oR3-P3-P4
1, and 2	skewness-R3-F3-F4, skewness-alpha2-Pz, kurtosis-gamma-mgamma-F3-F4, skewness-alpha-F3, skewness-gamma-mbeta-F4
1, and 3	mean-R2oR3-P3-P4, std-R2oR3-P3-P4, median-R2oR3-P3-P4, kurtosis-R2oR1-P3-P4, skewness-R2oR3-P3-P4, skewness-R2oR1-P3-P4
1, and 5	skewness-tab-F4
2, and 3	skewness-R2oR3-F3
2, and 5	skewness-R1-F3
3, and 4	skewness-delta-mdelta-P4, skewness-gamma-mgamma-F3-F4
3, and 5	skewness-theta-mdelta-P3, skewness-gamma-mtheta-P3-P4
4, and 5	skewness-msc-beta-Fz-Pz, skewness-R2oR3-F3-F4
	**Pearson**
1, and 5	skewness-R2oR3-Pz, skewness-R2oR1-P4
2, and 3	mean-msc-beta-P3-P4, skewness-beta-mbeta-P4, median-msc-beta-P3-P4
3, and 4	kurtosis-beta-mtheta-Cz
4, and 5	kurtosis-alpha1-F3-F4, skewness-R2oR3-Cz
	**Spearman**
1, and 2	kurtosis-R1-F3-F4
1, and 5	skewness-R2oR3-Pz, skewness-R2oR1-P4
3, and 4	std-alpha-mdelta-P3-P4, kurtosis-beta-mtheta-Cz, skewness-msc-delta-F3-P3
3, and 5	cv-msc-beta-F3-P3
4, and 5	kurtosis-alpha1-F3-F4

Table S3. Consistently Selected Features from Group 2 - MRI Features.

Runs	Elements
**MRMR**	
1, 2, 3, 4, and 5	aparc_area_lh_frontalpole_area, aparc_area_lh_superiortemporal_area
1, 2, 3, and 4	wm_rhCerebralWhiteMatterVol, aparc_area_lh_entorhinal_area, wm_Right_UnsegmentedWhiteMatter, wm_rh_insula
1, 2, 3, and 5	wm_EstimatedTotalIntraCranialVol, wm_lhCerebralWhiteMatterVol
1, 2, 4, and 5	wm_MaskVol
1, 3, 4, and 5	wm_rh_supramarginal, wm_rh_superiorparietal, wm_rh_frontalpole, wm_CerebralWhiteMatterVol, wm_rh_superiortemporal
2, 3, 4, and 5	wm_rh_transversetemporal
1, 3, and 4	wm_rh_rostralmiddlefrontal
1, 3, and 5	wm_rh_postcentral
3, 4, and 5	aseg_lhSurfaceHoles
1, and 2	wm_rh_inferiortemporal, aseg_3rd_Ventricle
1, and 3	wm_rh_superiorfrontal
1, and 5	wm_rh_posteriorcingulate
2, and 3	aparc_area_rh_lateraloccipital_area, wm_lh_pericalcarine
2, and 4	aparc_area_rh_transversetemporal_area, wm_Left_UnsegmentedWhiteMatter
4, and 5	wm_rh_rostralanteriorcingulate
**Pearson**	
2, 3, 4, and 5	aparc_area_rh_lateraloccipital_area, aparc_area_lh_lateraloccipital_area
1, 2, and 3	aparc_area_rh_transversetemporal_area
1, 2, and 4	aparc_area_lh_postcentral_area
1, 4, and 5	aparc_CT_rh_fusiform_thickness
2, 3, and 4	wm_rh_fusiform, wm_rh_lateraloccipital, aparc_CT_rh_rostralmiddlefrontal_thickness
1, and 2	wm_lh_postcentral, wm_lh_entorhinal, aparc_area_lh_entorhinal_area
1, and 3	aseg_Left_Putamen
1, and 4	aparc_area_rh_precuneus_area, aparc_area_rh_inferiortemporal_area
1, and 5	aparc_CT_rh_temporalpole_thickness
2, and 3	aparc_CT_lh_parahippocampal_thickness, wm_lh_lingual, aparc_area_lh_fusiform_area, aparc_area_lh_lingual_area
2, and 4	aparc_area_rh_fusiform_area, wm_lh_bankssts
3, and 5	aseg_Right_Hippocampus, aseg_SubCortGrayVol, wm_lh_inferiortemporal
4, and 5	wm_rh_bankssts, aseg_Right_Amygdala, aparc_CT_rh_insula_thickness
**Spearman**	
2, 3, 4, and 5	wm_rh_fusiform, wm_rh_lateraloccipital
1, 3, and 5	aseg_Left_Putamen, aseg_Right_Putamen
1, 4, and 5	aparc_CT_rh_fusiform_thickness
2, 3, and 5	aparc_area_rh_lateraloccipital_area, aparc_area_lh_lateraloccipital_area
1, and 2	aparc_area_rh_transversetemporal_area, wm_lh_entorhinal, aparc_area_lh_entorhinal_area
1, and 3	wm_rh_parstriangularis
1, and 4	aparc_area_lh_rostralmiddlefrontal_area, aparc_area_lh_bankssts_area, aparc_area_rh_caudalmiddlefrontal_area
1, and 5	wm_rh_inferiortemporal, aparc_CT_rh_temporalpole_thickness
2, and 3	wm_lh_lingual, aparc_area_lh_fusiform_area, aparc_area_lh_lingual_area
2, and 4	aparc_CT_rh_rostralmiddlefrontal_thickness, wm_lh_bankssts
3, and 5	aseg_SubCortGrayVol
4, and 5	wm_rh_bankssts, aparc_CT_rh_insula_thickness

Table S4. Consistently Selected Features from Group 3 - EEG and MRI Features.

Runs	Elements
	**MRMR**
1, 2, 3, 4, and 5	wm_EstimatedTotalIntraCranialVol, cv-alpha-mdelta-Cz, mean-alpha-mdelta-Cz
1, 2, 3, and 4	mean-gamma-mgamma-Fz
1, 2, 3, and 5	kurtosis-beta-mtheta-F3-F4
3, 4, and 5	cv-beta-mtheta-Pz
1, and 2	mean-gamma-mbeta-Cz, cv-alpha2-Fz, std-alpha-P3-P4, std-msc-gamma-Fz-Pz
1, and 5	std-R1-P4
2, and 3	std-gamma-mtheta-Pz
2, and 4	kurtosis-gamma-mdelta-Cz
	**Pearson**
1, and 2	aparc_area_lh_entorhinal_area
1, and 5	aparc_CT_rh_fusiform_thickness, skewness-R2oR1-P4
2, and 3	mean-msc-beta-P3-P4, aparc_area_rh_lateraloccipital_area, wm_rh_lateraloccipital, median-msc-beta-P3-P4
3, and 4	kurtosis-beta-mtheta-Cz
4, and 5	kurtosis-alpha1-F3-F4, skewness-R2oR3-Cz
	**Spearman**
1, and 2	kurtosis-R1-F3-F4
1, and 5	skewness-R2oR3-Pz, skewness-R2oR1-P4
3, and 4	std-alpha-mdelta-P3-P4, kurtosis-beta-mtheta-Cz, skewness-msc-delta-F3-P3
3, and 5	aseg_Left_Putamen, wm_rh_lateraloccipital, cv-msc-beta-F3-P3
4, and 5	kurtosis-alpha1-F3-F4

Table S5. Comparative regression performance from models formed by the features discussed in Tables S2, S3, and S4

Model	EEG	MRI	EEG + MRI
**SVM Linear**	1.986 ± 0.224	1.745 ± 0.183	1.825 ± 0.203
**SVM RBF**	1.816 ± 0.157	1.807 ± 0.154	1.769 ± 0.149
**Random Forest**	1.798 ± 0.176	1.715 ± 0.186	**1.682 ± 0.177**
**KNN**	1.993 ± 0.217	1.887 ± 0.181	1.892 ± 0.209
